# Staging resection of multiple primary esophageal cancer by endoscopic submucosal dissection and esophagectomy: A case report

**DOI:** 10.1097/MD.0000000000010657

**Published:** 2018-05-04

**Authors:** Yufeng Yao, Yimin Wu, Ying Chai

**Affiliations:** a Department of Thoracic Surgery, the Second Affiliated Hospital, College of Medicine, Zhejiang University; b Department of Thoracic Surgery, the First Affiliated Hospital of Zhejiang Chinese Medicine University, Hangzhou, China.

**Keywords:** endoscopic submucosal dissection, minimally invasive esophagectomy, multiple primary esophageal cancer

## Abstract

**Rationale:**

Multiple primary esophageal cancer pose great risks to patients and are always challenging to resect surgically. In order to reduce the risk of postoperative complication and meet the needs of minimally invasive and precision medicine, new treatment plans have been always developed for patients with multiple primary esophageal cancer.

**Patient concerns:**

A 75-year-old man was admitted to our hospital for aggravated dysphagia. No significant abnormalities were identified on physical examination.

**Diagnoses:**

Endoscopic examination detected 3 masses in the esophagus and biopsy confirmed multiple primary esophageal cancer.

**Intervention:**

The patient received a new staging treatment procedure firstly and an innovative single-position, minimally invasive Ivor Lewis esophagectomy in our hospital.

**Outcomes:**

This patient discharged one week after the surgery and enjoyed a good health during our follow up for 30 month.

**Lessons:**

We believe our procedure provides a beneficial new alternative approach for patients with multiple primary esophageal cancer.

## 1 Introduction

Surgical resection is the gold standard of treatment for localized esophageal cancer.^[1]^ Despite the presence of multiple primary cancers, curative surgeries are still very effective treatment.^[2]^ Traditional, multiple primary esophageal cancer is usually resected by esophagectomy. However, this procedure always makes the patients facing higher risk of severe postoperative complications, such as anastomotic fistula.^[3,4]^ Therefore, modified treatments that maintain a balance between radical tumorectomy and minimally invasive approaches should be considered. We herein provide the first report outlining a staging resection procedure for a 75-year-old male patient with multiple primary esophageal cancer by endoscopic submucosal dissection (ESD) and esophagectomy.

## 2 Case report

**Figure 1:**
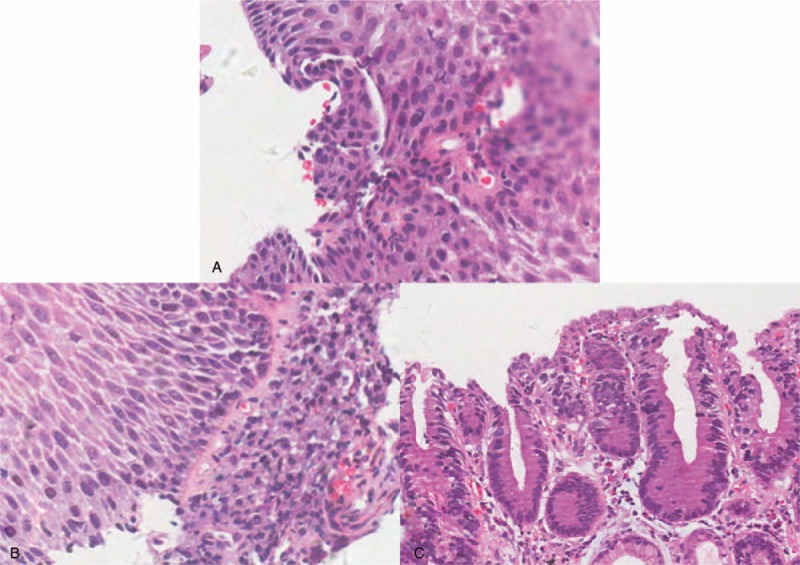
(A) (26 cm from the incisors): high grade intraepithelial neoplasia. (B) (40 cm from the incisors): canceration of esophageal squamous mucosa. (C) (cardia of stomach): low grade intraepithelial neoplasia. (hematoxylin and eosin, original magnification ×20).

A 75-year-old male patient was admitted to our institution because of aggravated dysphagia for 2 months. No significant abnormalities were identified on physical examination. No remarkable medical, family, and psychosocial history was observed. Endoscopic examination indicated a mass lesion 26 (foci A) cm from the incisors, one 40 cm from the incisors (foci B), and another in cardia of stomach (foci C). The results of biopsy showed the high grade intraepithelial neoplasia (foci A), canceration of esophageal squamous mucosa (foci B), and low grade intraepithelial neoplasia (foci C) (Fig. 1). These findings were supported by positron emission tomography-computed tomography studies which both supported the diagnosis of esophageal cancer without any metastatic lesions.

After Multi-Disciplinary Team discussion, we decide to perform a staging ESD combined with minimally invasive esophagectomy on this patient. The foci A plans to be resected by an ESD, and the foci B and C will be resected via an esophagectomy.

**Figure 2:**
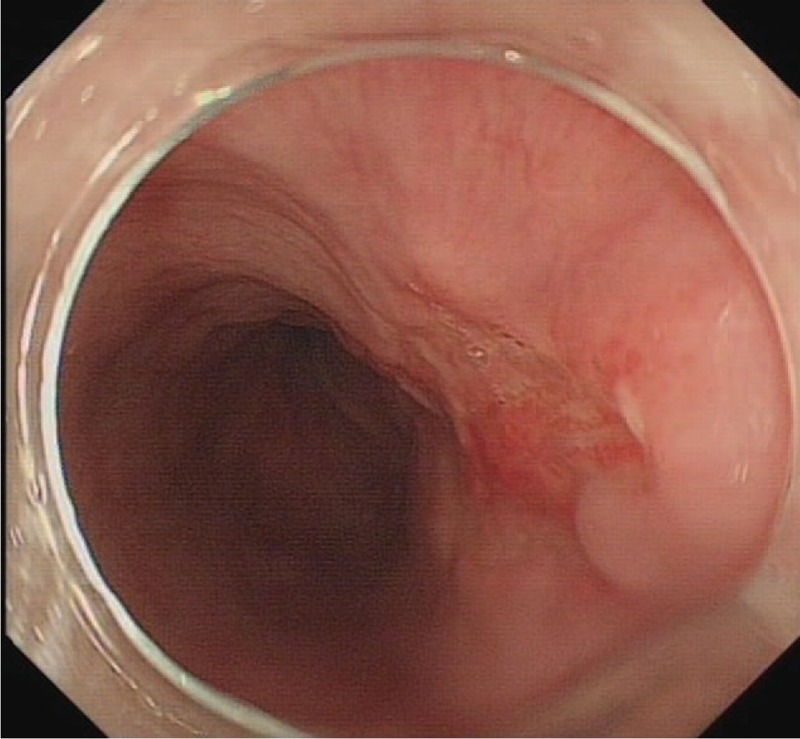
a II_c_ lesion was observed 26 cm from the incisors.

The foci A was completely removed through an ESD. During the surgery, we observed a II_c_ lesion 26 cm from the incisors (Fig. 2). The pathology confirmed the high grade intraepithelial neoplasia, which is the same as before.

**Figure 3:**
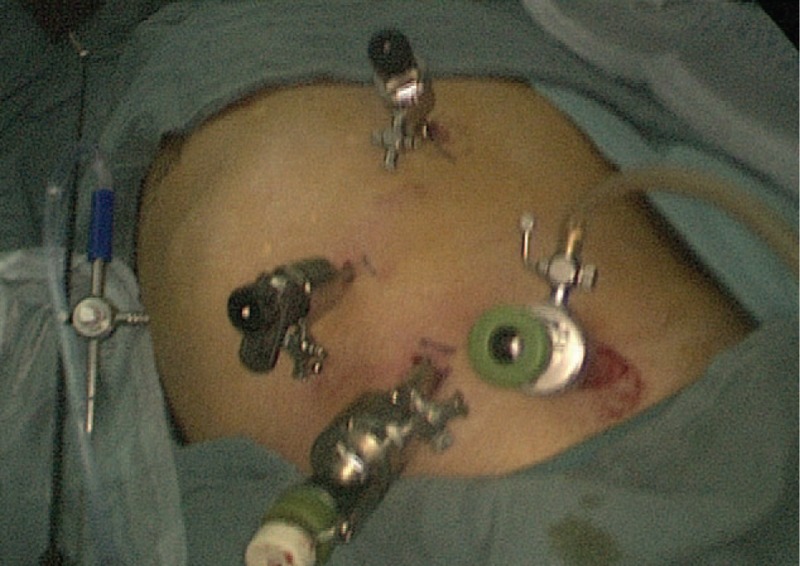
Location of 4 abdominal ports on the right side of the abdomen.

After rehabilitation of ESD, the patient underwent a single-position, minimally invasive Ivor Lewis esophagectomy. After general anesthesia and inserting a double-lumen endotracheal tube, the patient took the supine position and the right chest was elevated by 30°. Four abdominal ports are made on the right side of the abdomen (Fig. 3). After we detected the specific position of the tumor and confirmed the target lesion based on the preoperative imaging examination, the stomach lower esophagus was mobilized, and lymphadenectomy was performed. Afterwards, thoracic procedure was performed. A 5 cm incision was made in the 5th intercostal space, and then we mobilized the upper esophagus and retrieved lymph nodes in the same way. After the anvil head was inserted, the tumor was resected under the premise of ensuring the negative margin (3 cm) in the chest and the broken end of the esophagus was purse sutured. Then, a 5 cm median abdominal incision was made below the xiphoid. The stomach was brought out from this incision, then we removed the cardia and made a gastric tube in the vitro and brought it to the chest through the esophageal hiatus. Thoracic esophagogastric anastomosis was successful performed at last. After placement of a stomach tube and a nasogastric tube, all the incisions were then closed. The postoperative pathology confirmed esophageal squamous mucosa without lymph node metastasis. The postoperative course was uneventful. The patient discharged 1 week after the surgery and enjoyed a good health during our follow-up for 30 months.

## 3 Discussion

Esophagectomy is always the most effective treatment for localized esophageal cancers.^[5]^ However, outcomes of a simple esophagectomy on patients with multiple primary esophageal cancer are suboptimal. Because it always invade and colonize long segments of the esophagus, which means the necessity of a high esophagus-stomach anastomosis. It may lead increased risks of fistula and other complications after surgery in patients with multiple primary esophageal tumors.

Here, we report a patient diagnosed with localized esophageal cancer. However, this patient is very old and his nutritional status is very poor (preoperative BMI is 16.7). Furthermore, the foci A is just 26 cm from the incisors, we have to make a cervical anastomosis after esophagectomy. Those above are all high risk factors of anastomotic fistula and other postoperative complications.^[3,6,7]^ Therefore, we decided to find an approach that gives consideration to both minimally invasive techniques and tumor resection. Endoscopy revealed 3 lesions in the esophagus, among which the pathology of the foci A was confirmed to be high grade intraepithelial neoplasia. An ESD is the optimal choice for such precancerosis, which may achieve more minimal invasion than esophagus.^[5,8–10]^ The pathology of foci B showed the tumor infiltrated the submucosal layers. Therefore, an esophagectomy with curative intent is the best choice.^[5]^ Additionally, the lesion in the cardia of stomach can be resected at the same time because the cardia will be resected in order to make a gastric tube. This treatment strategy can reduce surgery invasive indeed and promote a completely resection of tumor.

Furthermore, in order to pursue minimally invasive approaches, we adopted a new surgical procedure named single-position, minimally invasive Ivor Lewis esophagectomy. Compare with other established minimally invasive procedures, this procedure only requires 1 position during surgery, the surgeon can extracorporeal perform stapling of the gastric conduit under direct vision, and the thoracoscopic anastomosis is created using a minilaparotomy and transhiatal approach.^[11]^ Undoubtedly, this procedure should substantially decrease the operation time and postoperative rehabilitation and hospitalization.

Based on our findings, this treatment strategy has demonstrated its feasibility in patients with multiple primary esophageal cancer. At present, this treatment is limited in patients with widely separated focuses in the esophagus and 1 lesion can be fully treated by ESD (less than 2 cm in diameter and invasion no deeper than the superficial submucosa).^[12]^ We believe our procedure is technically feasible and provides a promising alternative approach for the reconstruction of chest wall after resection.

## Author contributions

Drafted the manuscript and collect the date: Yimin Wu.

Collection and interpretation of the genomics date: Yufeng Yao.

Collect the date and modifying the manuscript: Ying Chai.

All authors read and approved the final manuscript.

**Data curation:** Yufeng Yao, Yimin Wu.

**Project administration:** Ying Chai.

**Writing – original draft:** Yufeng Yao.

**Writing – review & editing:** Yimin Wu, Ying Chai.
